# Enhanced photovoltaic properties in dye sensitized solar cells by surface treatment of SnO_2_ photoanodes

**DOI:** 10.1038/srep23312

**Published:** 2016-03-18

**Authors:** Kaustubh Basu, Daniele Benetti, Haiguang Zhao, Lei Jin, Fiorenzo Vetrone, Alberto Vomiero, Federico Rosei

**Affiliations:** 1 Institut National de la Recherche Scientifique-Énergie, Matériaux et Télécommunications, Université du Québec, Varennes, QC, Canada; 2 Department of Engineering Sciences and Mathematics, Luleå University of Technology, 971 98, Luleå, Sweden; 3 Institute for Fundamental and Frontier Science, University of Electronic Science and Technology of China, Chengdu 610054, P.R. China; 4 Centre for Self-Assembled Chemical Structures, McGill University, Montreal, QC, Canada

## Abstract

We report the fabrication and testing of dye sensitized solar cells (DSSC) based on tin oxide (SnO_2_) particles of average size ~20 nm. Fluorine-doped tin oxide (FTO) conducting glass substrates were treated with TiO_x_ or TiCl_4_ precursor solutions to create a blocking layer before tape casting the SnO_2_ mesoporous anode. In addition, SnO_2_ photoelectrodes were treated with the same precursor solutions to deposit a TiO_2_ passivating layer covering the SnO_2_ particles. We found that the modification enhances the short circuit current, open-circuit voltage and fill factor, leading to nearly 2-fold increase in power conversion efficiency, from 1.48% without any treatment, to 2.85% achieved with TiCl_4_ treatment. The superior photovoltaic performance of the DSSCs assembled with modified photoanode is attributed to enhanced electron lifetime and suppression of electron recombination to the electrolyte, as confirmed by electrochemical impedance spectroscopy (EIS) carried out under dark condition. These results indicate that modification of the FTO and SnO_2_ anode by titania can play a major role in maximizing the photo conversion efficiency.

Over the last two decades, dye sensitized solar cells (DSSCs) have been widely explored as potential alternatives to conventional silicon photovoltaic (PV) devices due to their low cost, abundance of raw material, facile fabrication process and overall good photovoltaic performance (record efficiency above 14%)^1^,^2^. Compared to typical semiconductors used in p-n junction solar cells, the materials employed in a DSSC photoanode may have lower purities^3^, thereby leading to lower production costs^4^,^5^. However, to become a realistic candidate to replace traditional solar cells, several challenges have to be addressed to enhance device performance. The photoanode material plays a major role for such purposes: TiO_2_ nanoparticle films have been widely used in DSSCs, thanks to the very fast electron injection rates from the excited state of the dye into the TiO_2_ nanoparticle conduction band, of the order of femtoseconds. On the other hand, due to limited electron mobility in TiO_2_, the high electron recombination rate leads to degradation of photoconversion efficiency (PCE)^6^.

Other potentially suitable semiconducting metal oxides were recently considered, such as ZnO and SnO_2_^7^,^8^,^9^,^10^. These are appealing candidate materials, due to their higher electron mobility, as compared to TiO_2_^11^,^12^ and specific advantages, such as: (i) the ZnO band gap and band positioning is energetically similar to TiO_2_^1^; (ii) SnO_2_ on the other hand is a promising oxide material because of its higher electronic mobility and large band gap (3.8 eV)^13^. The mobilities reported in both single crystal SnO_2_^14^ as well as nanostructures^15^ are orders of magnitude higher than in single crystal TiO_2_^6^. The high electron mobility in SnO_2_ compared to its counterpart TiO_2_, leads to faster diffusion-mediated transport of photoinjected electrons. The wider bandgap of SnO_2_, compared to anatase TiO_2_ (3.2 eV) creates fewer oxidative holes in the valence band under UV illumination, so as to minimize dye degradation rate and long term stability of DSSCs^16^,^17^. Because of these appealing properties, SnO_2_ has been recently utilized in other advanced applications^18^,^19^,^20^, including hydrogen gas sensing^21^,^22^. Various synthesis techniques of SnO_2_ nanocomposites have also been reported in recent literature^23^,^24^. In the specific field of DSSCs, a drawback of SnO_2_ is its faster electron recombination kinetics and lower trapping density compared to TiO_2_, resulting from a 300 mV positive shift of conduction band relative to TiO_2_, which results in an increased dark current, thereby limiting the open circuit voltage^25^,^26^. In addition, SnO_2_ has a lower isoelectric point (IEP, at pH 4–5) than anatase TiO_2_ (IEP at pH 6–7)^27^, which leads to lower dye adsorption with acidic carboxyl groups^26^, decreasing the optical density of the photoanode and its ability to absorb solar radiation. In DSSCs, the FTO glass can directly come in contact with the electrolyte, since the mesoporous photoanode cannot uniformly cover the entire FTO surface. At the FTO-electrolyte interface, charge recombination limits electron collection and affects PCE^28^,^29^,^30^,^31^,^32^. Modification of the FTO/electrolyte interface plays a key role in boosting DSSC performance by suppressing the recombination of electrons from the FTO to the electrolyte. A promising approach for such interface modification is to add a compact metal oxide blocking layer on FTO that would inhibit back electron transport from FTO to the electrolyte^33^. Also, TiO_2_-SnO_2_ based heterojunctions can be potentially considered to mitigate the problem of low electron mobility in TiO_2_ and fast electron recombination in SnO_2_. In fact, the conduction band edge of SnO_2_ is at a lower position than that of TiO_2_, so the electrons injected from the dye to TiO_2_ can be efficiently injected into SnO_2_, which in turn also has a higher mobility, so that the overall electron conductivity of the system can be enhanced^13^. The TiO_2_-SnO_2_ heterojunction creates a cascading band structure when layered with the N719 dye^34^,^35^. Recently, a new record conversion efficiency around 7.4% has been set with SnO_2_ multiporous nanofibers DSSC with TiCl_4_ treatment by Qamar Wali and co-workers^19^. Multistep electron transport down a cascading band structure increases carrier lifetime and reduces charge recombination^36^. Also, a thin TiO_2_ passivating layer at the surface of SnO_2_ nanoparticles can reduce recombination of electrons from the SnO_2_ photoanode to the electrolyte, which is one of the main contributors that cause poor performance of SnO_2_ solar cells.

In this manuscript, we implement a modified SnO_2_ photoanode, in which the concept of efficient electron injection is pursued in a SnO_2_-TiO_2_ heterojunction (Fig. 1), to form a cascading band structure^34^,^35^ by modifying the SnO_2_ photoanode with TiCl_4_ or TiO_x_ precursor solution, and treating the FTO substrates with the same precursor solutions to form a blocking layer. The morphological characterization of the photoanode indicates an interconnected structure of SnO_2_ and TiO_2_ nanoparticles, where small TiO_2_ nanoparticles grow on top of each other forming wire-like structures connecting the mesoporous SnO_2_ photoanode. The functional characterization of the entire solar cell (which demonstrate a marked improvement of photovoltaic parameters) indicated an overall increase of ~90% in the PCE from 1.48% without any treatment, to 2.85% achieved with TiCl_4_ treatment.

## Results and Discussion

Size tuning of nanoparticles is very important in determining the performance of DSSCs. The optimum size is around 20 nm, because for larger nanoparticles, less surface per unit volume is available for dye adsorption. On the other hand, in the case of smaller sized nanoparticles, a larger number of grain boundaries can be found, which increases the probability of electron trapping^37^,^38^,^39^,^40^, and smaller pore size, which can inhibit dye penetration during the sensitization process^41^. We chose SnO_2_ nanoparticles ∼20 nm in diameter for photoanode preparation. The surface morphology of different photoanodes, with/without treatment is illustrated in Fig. 2(a–d). The typical mesoporous structure is visible after sintering. The TiCl_4_ treatment (Fig. 2b,c) induces the formation of small nanoparticles of about 5 nanometers (nm), which are confirmed to be composed of titanium oxide by energy dispersive X-ray spectroscopy (EDX) selectively performed on a single nanoparticle, as shown in Fig. 3. The TiO_2_ nanoparticles preferentially form short interconnected nanowires (Fig. 2b,c). The higher the TiCl_4_ molar concentration, the more connected is the wire-like structure (see comparison of Fig. 2b,c and Fig. 2f,g). The TiO_x_ treatment induces the formation of completely different structures compared to TiCl_4_: the TiO_2_ structures seem to cover the larger SnO_2_ particles. If we compare the scanning electron microscope (SEM) and transmission electron microscope (TEM) images from Fig. 2, we can more clearly observe the influence of the different precursors on bare SnO_2_ nanoparticles. Inspection of TEM images reveal that the pure SnO_2_ sample consists of spherical nanoparticles whose average size is about 20 nm (Fig. 2e). However, TEM images Fig. 2f through Fig. 2h indicate that spherical nanoparticles of size around 20 nm are decorated with smaller size nanoparticles, which sometimes grow on top of one another to form nanowire like structures (SEM images 2b and 2c) and sometimes cover the SnO_2_ nanoparticles (Fig. 2d). EDX mapping analysis is reported in Fig. 2. The elemental characterization of the SnO_2_ photoanode is shown in Fig. 2i. EDX carried out on SnO_2_ mesoporous photoanode treated with 50 mM TiCl_4_, 90 mM TiCl_4_ and TiO_X_ are shown in Fig. 2j, k and l, repectively and confirm the presence of the constituent elements of SnO_2_ and TiO_2_. High Resolution Transmission Electron Microscopy (HRTEM) was carried out on treated samples to examine their fine structure. The results are reported in Fig. 3, which shows the crystalline structure of the SnO_2_ and the TiO_2_ nanoparticles. Distinct lattice fringes in the HRTEM images highlight the presence of crystalline phases for both SnO_2_ (green coloured text) and TiO_2_ (red coloured text). HRTEM indicates that the crystalline phase of both SnO_2_ and TiO_2_ can be indexed to a rutile structure, (JCPDS No. 00-041-1445 and JCPDS No. 01-088-1175, respectively). This finding is consistent for both TiCl_4_ and TiO_x_ treatments. X-ray diffraction (XRD) analysis was performed to further confirm the crystalline structure of our anodes and is reported in Fig. S1. In all the cases the diffraction pattern matches with rutile SnO_2_ (JCPDS No. 00-041-1445). XRD reveals that the crystal structure of SnO_2_ nanoparticles did not change after TiO_x_ treatment or after the photoanalysis process. TiO_2_ peaks could not be found for the treated samples since the quantity is most likely below the detection limit.

**Figure 1:**
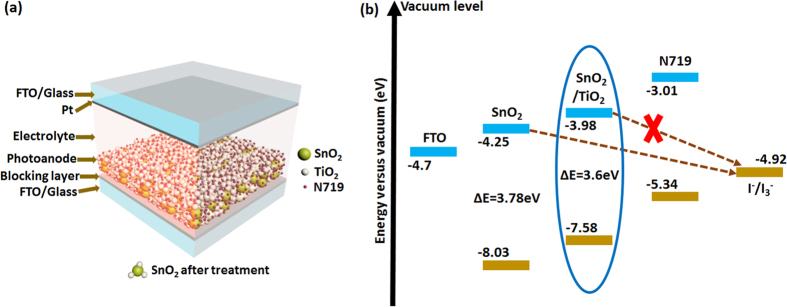
(**a**) Proposed schematic representation of DSSC. (**b**) Electronic band diagram showing the beneficial effect of the present methodology to modify SnO_2_ anode by TiO_2_ (encircled) in alleviating charge recombination from anode to electrolyte as opposed to only using SnO_2_ anode.The conduction band, valence band positions and band gap values of present SnO_2_ anode and SnO_2_ anode modified by TiO_2_ are calculated from data acquired from the combination of UPS and diffuse reflectance spectroscopy. All other values are from reported literature^61^,^62^,^63^.

**Figure 2:**
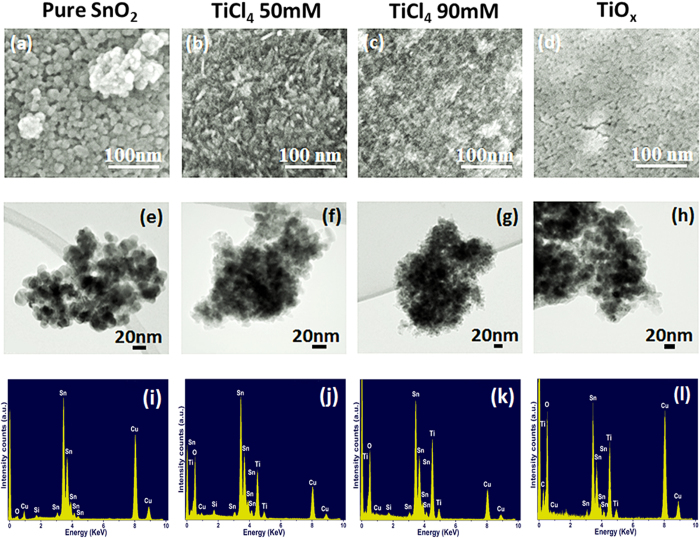
SEM images (**a–d**), TEM images (**e–h**), and EDX spectra (**i–l**) on different SnO_2_ photoanodes, confirming the presence of the constituent elements, SnO_2_ anodes: (**a,e,i**) before treatment; (**b,f,j**) treated with 50 mM TiCl_4_; (**c,g,k**) treated with 90 mM TiCl_4_; (**d,h,l**) treated with TiO_X_.

**Figure 3:**
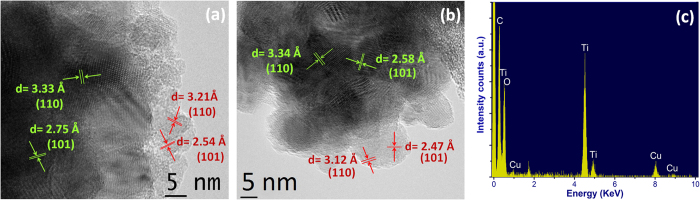
HRTEM images of (**a**) TiCl_4_ treated and (**b**) TiO_x_ treated SnO_2_ nanoparticles. (**c**) EDX spectra confirming the presence of constituent elements of TiO_2_ when selectively done on the TiO_2_ nanoparticles covering the SnO_2_ nanoparticles, the presence of Cu and C is due to the grid used as sample holder.

**Figure 4:**
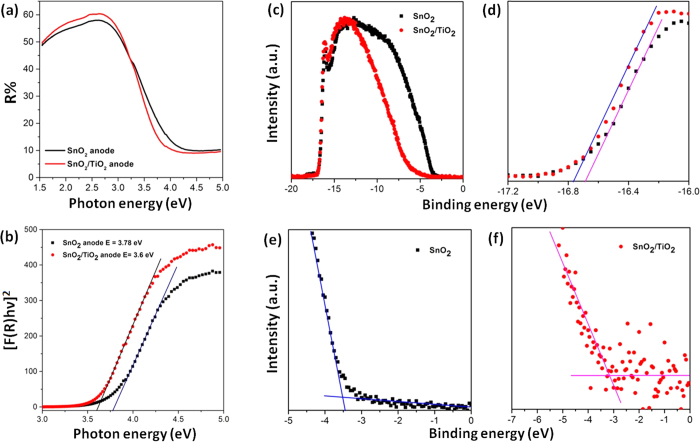
(**a**) Diffuse Reflectance spectra plotted with respect to photon energy for SnO_2_ anode and SnO_2_ anode after TiO_x_ post-treatment; (**b**) Kubelka-Munk transformed spectra for SnO_2_ anode and SnO_2_ anode after treatment; (**c**) Complete UPS of SnO_2_ and treated SnO_2_ anodes; (**d**) magnified view of high binding energy cut-off (Fermi level) and (**e,f**) low binding energy cut-off (VB edge) of SnO_2_ anode and SnO_2_ anode after TiO_x_ post-treatment respectively.

To determine the band gap of SnO_2_ photoanode before and after post-treatment, diffuse reflectance spectroscopy measurement were carried out. Kubelka Munk function was used to calculate the band gap of SnO_2_ photoanode before and after TiO_x_ post-treatment (Fig. 4). The band gap of SnO_2_ photoanode was found to be 3.78 eV, which is consistent with literature^37^ and the band gap of SnO_2_ photoanode after TiO_x_ post-treatment was found to be 3.6 eV. Ultraviolet Photoelectron spectroscopy (UPS) analysis (Fig. 4c–f), was performed to identify the band positions of the various photoanodes. The valence band (VB) edge for SnO_2_ anode is −8.03 eV, which shifts to a higher value of −7.58 eV after TiO_x_ post-treatment. The energy band gap values from Kubelka-Munk transformed diffuse reflectance spectra for SnO_2_ anode and SnO_2_ anode after TiO_x_ post-treatment along with values of valence band edge from UPS measurement reveals the lowest position of the conduction band (CB) for both the samples. The values of CB minima, VB maxima, and the band gap for SnO_2_ anode and SnO_2_ anode after TiO_x_ post-treatment are reported in Fig. 1b. X-ray photoelectron spectroscopy analysis (XPS), was performed to detect any possible shift in the binding energy for the Ti 2p and Sn 3d peaks. The high resolution XPS of Ti 2p of the treated anode and the treated anode after photoanalysis process (Fig. S2) shows that there is no significant change for Ti 2p peaks after the cell operation, which means that the chemical state of Ti^4+^ does not change during the photoanalysis process. Similar inference can be also drawn in the case of Sn 3d peaks, which are reported in Fig. S3, confirming that the chemical state of Sn^4+^ does not change.

**Figure 5:**
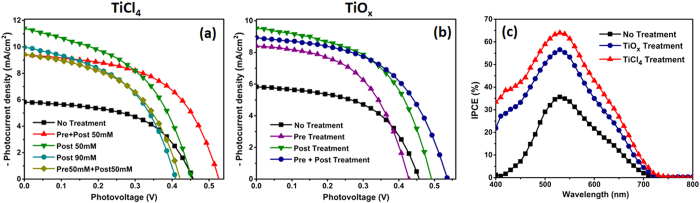
(**a**) Photocurrent density–photovoltage (J-V) characteristics of SnO_2_ nanoparticle based DSSCs, under 1 Sun illumination, obtained from pre- and post-treatment of different concentration of TiCl_4_ precursor; (**b**) J-V characteristics obtained from pre- and post-treatment of TiO_x_ precursor; (**c**) Dependence of absolute IPCE of the solar cells with dye-sensitized SnO_2_ nanoparticle electrodes modified with TiO_X_ and TiCl_4_ tretament.

To understand the influence of the different treatments on the cell performance, the functional properties of the solar cells were investigated under standard AM1.5G simulated sun irradiation. The results are presented in Fig. 5, and the PV parameters (open circuit voltage (*V*_oc_), short circuit photocurrent density (*J*_sc_), fill factor (FF), and PCE) are shown in Table 1.A maximum PCE of 2.85% is achieved in the TiCl_4_-treated cell (with pre- and post-treatment) and 2.66% for TiO_x_ pre- and post-treatment, compared to 1.48% for the pristine SnO_2_ photoanode. This marked improvement in the PCE originates from the higher *V*_oc_ and *J*_sc_ of the cells, which in turn are induced by the treatments. A possible explanation in the increase of *V*_oc_ can be due to an increase of electron density in SnO_2_ as a consequence of effectively suppressed recombination, the Fermi level shifts to a more negative value^42^ and since *V*_oc_ is the difference between the Fermi level in the semiconductor and the redox potential of I–/I^3−^ of the electrolyte, a higher value of open circuit voltage is recorded for all treated anodes. A high *V*_oc_ of 0.53 V can be observed for the best TiCl_4_ and TiO_x_ treated cells. Also due to the treatment, the electronic contact between the nanoparticles is improved because of broadened contact interface and the increased contact point which make the electrons traverse the film more easily^43^, which further confirms the reduction of recombination. In addition, interconnection between the nanoparticles facilitate the percolation of electrons from one nanoparticle to other and lead to a global increase of current^43^. However, when we introduce additional TiO_2_ by treating our anodes with higher molar concentration of TiCl_4_, due to the increase in nucleation and growth of nanoparticles, which leads to higher concentration and density of the nanoparticles, there is a reduction of film porosity, which may lead to a decrease of dye adsorption, thus deteriorating the PV properties^44^,^45^. For TiCl_4_ modification, the J_sc_ increases by ∼60% from the untreated cell and for TiO_x_ modification, an increase of 53% is observed. The family of IPCE curves in Fig. 5 are constructed with the IPCE spectra of a normal SnO_2_ photoanode cell and the best cells with both TiO_x_ and TiCl_4_ treatment. The curves are in accordance with the current density versus voltage curves where we observe a marked improvement as a result of treatment. As already established from SEM images, in case of TiCl_4_ treatment, the small titania nanoparticles connect with each other, forming nanowire like structures. On the other hand, in the case of TiO_x_ treatment, TiO_2_ particles cover the SnO_2_, effectively reducing direct contact of SnO_2_ with the electrolyte, possibly decreasing recombination, thereby leading to an enhancement of IPCE.

**Figure 6:**
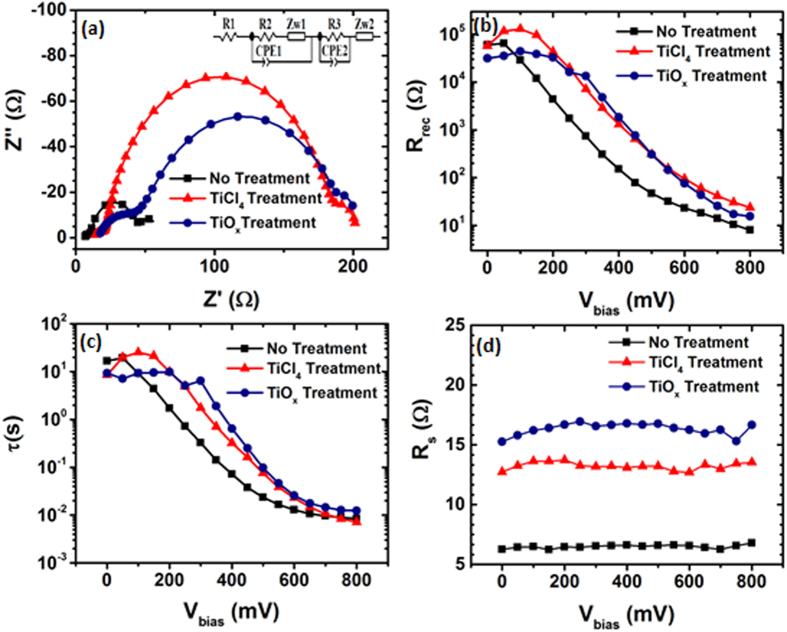
(**a**) Nyquist plots of devices with and without treatment, under dark condition and around V_oc_. The inset shows the model used for fitting the data. From left to right of the model in the inset: series resistance (R1), electron transport and back reaction at the TiO_2_/electrolyte interface, charge transfer at electrolyte/Pt-FTO interface and diffusion of ions in the electrolyte. (**b**) Recombination resistance as calculated from EIS analysis. (**c**) Electron lifetime obtained from the product R_rec_*C_μ_. (**d**) Series resistance calculated from EIS analysis.

We performed electrochemical impedance spectroscopy (EIS) analysis to understand the physical-chemical processes which regulate charge dynamics in our cells. Fig. 6 displays the EIS spectra under dark condition of our pristine SnO_2_ cell and the two best cells after TiCl_4_ and TiO_x_ treatments. The treated cells were subjected to a pre-treatment that forms a blocking layer (BL) and a post-treatment that influenced film morphology and PV performances^3^,^46^,^47^. The role of the BL in DSSCs is still under debate; different hypotheses have been formulated. Cameron and Peter, and more recently Park and co-workers demonstrated the ability of the BL to prevent back reaction (i.e. electron recombination from FTO to electrolyte) of electrons and attributed the improved performance to the charge transfer resistance at the BL/electrolyte interface. Hence, BL was effective in reducing charge losses at the FTO/anode interface^48^,^49^. On the other hand, Fabregat and co-workers indirectly deduced that the main factor leading to the enhancement in PCE was the improved physical contact between the coated FTO and the TiO_2_ film^50^. Fig. 6a compares the Nyquist plots of three different cells (bare SnO_2_, TiCl_4_ and TiO_x_ treatment) under dark condition and at bias voltage equals to *V*_oc_. Three semicircles are clearly visible: the high frequency arc is attributed to the redox reaction at the platinum counter electrode, the medium arc takes in account the oxide/electrolyte interface and the low frequency arc is related to the Nerst diffusion in the electrolyte^51^,^52^. The medium arc is evidently larger for the treated cells, indicating that the methods used, increase the resistance, thus lowering the recombination processes at the oxide/electrolyte interface. Fig. 6b shows the trend of the recombination resistance (*R*_rec_) as a function of applied bias. For both treatments, the values of *R*_rec_ are increased compared to the pristine cell, thus indicating reduced recombination after treatment. The overall trend of *R*_rec_ clearly shows that after the treatment, the recombination resistance is increased across the whole bias range, confirming a decrease in recombination phenomena. The region at low bias (below ~250 mV) is dominated by the charge recombination and capacitance of the back layer^53^,^54^. In this region it is thus possible to observe the different contributions to the recombination resistance due to different back layer (in fact, with and without the blocking layers, the response of the system will be different: without BL, increased FTO/electrolyte back reaction occurs)^48^,^55^. In our case, we can observe that *R*_rec_ increases, in particular for the TiCl_4_ treatment, indicating the effectiveness of the BL to reduce charge recombination at this interface. The increase in *R*_rec_ for TiO_x_ treatment is lower compared to the TiCl_4_, probably due to presence of more voids in the blocking layer, which allow the electrolyte to come in contact with the FTO^56^.

In general, the combined effect of the pre- and post-treatment on the cells is an increase of the electrons lifetime, as shown in Fig. 6c: the lifetime is systematically higher for the treated cells. Similar results can be found in the literature where the presence of the BL suppresses the charge recombination at the FTO/electrolyte interface^49^,^57^,^58^. The main contribution of the post treatment is more difficult to observe in the EIS spectra, because this kind of treatment usually affects the morphology of the sample, modifying, for example, the total surface area. This in turn favors a higher dye loading and greater light harvesting^46^, yet has less influence on the electronic processes related to charge transport and recombination. As already found by Fabregat and co-workers, the treated cells present a higher value of the series resistance (*R*_s_) in Fig. 6d, which is detrimental for the overall PCE of the cell^50^. However, this effect is largely compensated by a much higher reduction of the charge recombination at the FTO/electrolyte interface, so that the net result is the enhancement of PCE.

## Conclusions and Perspectives

In summary, we demonstrated the synthesis of SnO_2_ photoelectrodes prepared with SnO_2_ nanoparticles of average size 20 nm, treated with TiCl_4_ precursor solution and TiO_x_ flat film precursor solution. The modified photoanodes exhibit a significant relative increase in power conversion efficiency of the solar cell, which is attributed to the enhancement of functional properties of the DSSC. The blocking layer inhibits the transport of electrons back to the electrolytes, which are collected by the FTO, while passivation of the SnO_2_ mesoporous layer blocks the back-reaction pathway of photoinjected electrons from semiconductor anode to electrolyte. The enhancement of open circuit voltage was made possible due to the modification of the electronic band alignment because of the reduced charge recombination. The effect of interconnection of the electrode nanoparticles provide a better path for electron flow and plays a major role in enhancing the short circuit current of the device. The increase of R_rec_, and higher lifetime for solar cells with treated anodes further confirms reduced recombination and greater diffusion length of an electron before recombination, thus enhancing the functional properties of the DSSC, which led to nearly a 2-fold improvement in overall PCE.

We obtained experimental evidence (through UPS and diffuse reflectance) of the possibility to modify the electronic band structure in SnO_2_ photoanodes, which facilitates efficient electron transport in treated photoanodes, by suppressing charge recombination and thus improving the functional properties of the DSSC.

## Experimental Methods

### Preparation of SnO_2_ paste

The SnO_2_ powder used consists of nanoparticles of average particle size ~20 nm (American Elements 99.9% tin(IV) oxide). 1 g SnO_2_ powder was mixed with 5 ml ethanol as a solvent, 1 ml alpha-terpineol as dispersant, 0.5 g ethyl cellulose which acts as a thickener and 1 ml of water. The mixing of these ingredients were carried out in a beaker under overnight magnetic stirring. The solvent was removed by connecting it to a pump while continuous magnetic stirring until the volume of the mixture reduced to half of the starting volume.

### Pre- and post-treatment of the SO_2_ photoanode

The pre-treatment of the SnO_2_ photoanode, i.e. a blocking layer with TiO_2_ was deposited by spin coating the TiO_x_ flat film precursor solution at 2,000 r.p.m. for 60 s and subsequently heating at 500 °C for 30 min^59^. Alternatively, pre-treatment was also carried out by soaking the FTO substrates in 50 mM TiCl_4_ aqueous solution and 90 mM TiCl_4_ aqueous solution for 30 min at 70 °C, then they were flushed with deionized water and sintered at 450 °C for 30 min^3^. For post treatment of the SnO_2_ film, the same procedure as pretreatment was followed with the TiO_x_ and TiCl_4_ precursor solutions.

### Diffuse reflectance spectroscopy

Diffuse reflectance spectroscopy of SnO_2_ photoanode, and SnO_2_ photoanode treated with TiO_x_ flat film precursor solution was carried out using UV-Visible-NIR spectrometer, Perkin Elmer, Lambda 750 in the wavelength range of 250–800 nm with resolution of 2 nm. Kubelka Munk (K-M) function, was calculated following literature procedure^60^, and was plotted as a function of photon energy. K-M function was used to determine the band gap of SnO_2_ photoanode before and after post-treatment.

### Ultraviolet Photoelectron Spectroscopy

SnO_2_ photoanode, and SnO_2_ photoanode treaed with TiO_x_ precursor solution were studied with Ultraviolet Photoelectron Spectroscopy (UPS) collected using VG ESCALAB 3 Mark II high vacuum system. During the UPS measurement, illumination at 21.21 eV was provided by the He (I) emission line from a helium discharge lamp. Cutoff energies were determined from the intersection of a linear extrapolation of the cutoff region to a linear extrapolation of the baseline.

### DSSC assembly

Fluorine doped tin oxide (FTO) coated glass substrates (Pilkington, bought from Hartford Glass Co. Inc., USA) with sheet resistance 15 Ω/□ were sonicated for 30 min with 2% Triton X-100 in deionized water to remove traces of glue from packaging, then they were cleaned by 30 min sonication in isopropyl alcohol and then thoroughly rinsed with deionized water and dried in a filtered air stream. Subsequently a blocking layer with TiO_2_ was deposited using the techniques described above. Then, a layer of the prepared SnO_2_ paste was deposited using a simple doctor blade technique and dried in air for 10–15 min and then fired on a hot plate at 150 °C for 6 min. When the substrates cooled down, another layer was doctor bladed, followed by annealing at 500 °C for 30 min in air and then cooled back to room temperature. Then the SnO_2_ film was coated with a passivating layer of TiO_2_ by methods described already in the experimental methods section of Pre- and Post-Treatment of the SnO_2_ Photoanode. The treated anodes were then sensitized by dye, upon immersion in a 0.5 mM ethanolic solution of the commercial N719 dye (Ruthenizer 535-bisTBA from Solaronix) for 18 h and then washed with ethanol to remove any excess of unabsorbed dye molecules. Solar cells were assembled using a sputtered 10 nm thick platinized counter electrode on FTO glass substrate, 25 μm thick spacer Meltonix 1170-25 (to prevent the cell from short circuiting when the working and counter electrodes are clamped together), and iodide/tri-iodide redox couple HI-30as the electrolyte.

### PV measurements

Photocurrent density-voltage (J-V) characteristics were measured under simulated sunlight (1 sun = AM 1.5 G, 100 mW/cm^2^) using a Class AAA Solar Simulator from Photo Emission Tech (SS50AAA) with a Keithley 2400 sourcemeter. The simulator used has a class as per ASTM E927, AAA with non-uniformity of Irradiance of 2% or lower over 2 × 2 inch area. The system for device characterization was calibrated with a Si reference diode. The sourcemeter was controlled by a computer using an application written under TESTPOINT software platform. The fill factor (FF) and the power conversion efficiency (PCE) were calculated from the following equations^2^.


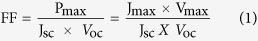



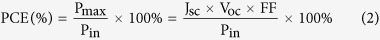


where P_max_ is the maximum power output, J_sc_ is the short-circuit current density, V_oc_ is the open-circuit voltage, J_max_ is the current density and and V_max_ is the voltage at maximum power output in the J-V curves, and P_in_ is the incident light power.

For the external quantum efficiency (EQE) or incident photon to current efficiency (IPCE) measurements of our devices, we used the Oriel IQE-200 certified system, which is calibrated using NREL certified Si and Ge detectors. The solar cells were connected using a probe station.

### EIS measurements

Electrochemical impedance spectroscopy (EIS) was studied under dark ambience using a SOLARTRON 1260A Impedance/Gain-Phase Analyzer. All spectra were collected by applying an external bias between 0 and 800 mV, in the 100 mHz-300 kHz frequency range. The measurements were carried out inside a Faraday cage for all the samples.

### Materials characterization

The morphology of the samples were studied using Scanning Electron Microscopy (SEM) and Transmission Electron Microscopy (TEM). SEM images were recorded using JEOL JSM840 at an accelerating voltage of 10 kV. Bright field TEM and high resolution HR-TEM imaging at 200 kV and energy dispersive X-ray spectroscopy (EDS) were carried out using JEOL JEM-2100F.

## Additional Information

**How to cite this article**: Basu, K. *et al.* Enhanced photovoltaic properties in dye sensitized solar cells by surface treatment of SnO_2_ photoanodes. *Sci. Rep.* **6**, 23312; doi: 10.1038/srep23312 (2016).

## Supplementary Material

Supplementary Information

## Figures and Tables

**Table 1:** Photovoltaic properties of DSSC with different configurations of TiO_x_ and TiCl_4_ treatment.

DSSC	Treatment type	V_oc_ (V)	J_sc_ (mA/cm^2^)	Fill Factor (FF)	Efficiency (%)
SnO_2__1	No Treatment	0.45	5.83	0.56	1.48
TiCl_4__1	TiCl_4_ Pre 50 mM + Post 50 mM	0.53	9.43	0.57	2.85
TiCl_4__2	TiCl_4_ Pre 50 mM + Post 90 mM	0.43	9.44	0.53	2.15
TiCl_4__3	TiCl_4_ Post 50 mM	0.45	11.4	0.51	2.64
TiCl_4__4	TiCl_4_ Post 90 mM	0.41	9.98	0.52	2.13
TiO_x__1	TiO_x_ Pre Treatment	0.43	8.41	0.54	1.96
TiO_x__2	TiO_x_ Pre + Post Treatment	0.53	8.94	0.56	2.66
TiO_x__3	TiO_x_ Post Treatment	0.49	9.52	0.55	2.59
