# Supplementary material for: Enhanced photovoltaic properties in dye sensitized solar cells by surface treatment of SnO2 photoanodes
Source: Sci Rep. 2016 Mar 18;6:23312. doi: 10.1038/srep23312 (PMC4796906; doi:10.1038/srep23312)
Supplement: Supplementary Information [file srep23312-s1.doc]

**Supplementary Information**

**Enhanced photovoltaic properties in dye sensitized solar cells by surface treatment of SnO_2_ photoanodes**

Kaustubh Basu^1^, Daniele Benetti^1^, Haiguang Zhao^1^, Lei Jin^1^, Fiorenzo Vetrone^1,4^, Alberto Vomiero^2*^, Federico Rosei^1,3,4*^

^1^ Institut National de la Recherche Scientifique - Énergie, Matériaux et Télécommunications, Université du Québec, Varennes, QC, Canada

^2^ Department of Engineering Sciences and Mathematics, Luleå University of Technology, 971 98, Luleå, Sweden

^3^ Institute for Fundamental and Frontier Science, University of Electronic Science and Technology of China, Chengdu 610054, PR China

^4^ Centre for Self-Assembled Chemical Structures, McGill University, Montreal, QC, Canada

**1. X-ray diffraction Analysis** **(XRD)**

XRD analysis has been performed using Bruker D8 Advance X-ray diffractometer for the SnO_2_ nanoparticle powder sample, SnO_2_ photoanode, SnO_2_ photoanode after TiO_x_ post-treatment, SnO_2_ photoanode after photoanalysis process, and SnO_2_ photoanode with TiO_x_ post-treatment after photoanalysis process reported in Figure S1. In all cases the diffraction pattern matches with rutile SnO_2_ (JCPDS No. 00-041-1445). XRD reveals that the crystal structure of SnO_2_ nanoparticles did not change after the treatment or the photoanalysis process. TiO_2_ peaks could not be found for the treated samples since the quantity is possibly below XRD detection limit. However TiO_2_ rutile structure corresponding to JCPDS card no. 01-088-1175 was confirmed from HRTEM images shown in Figure 3 in our manuscript.


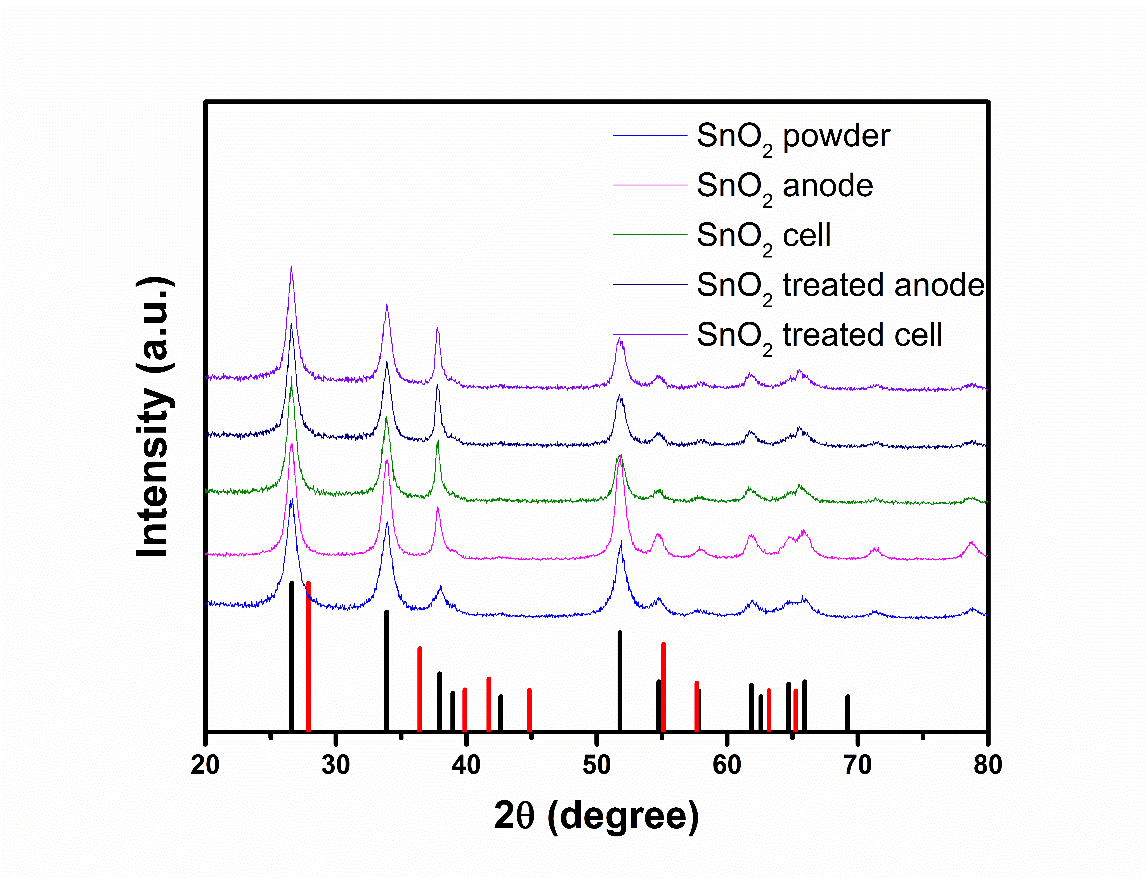


Figure S1: X-ray diffraction patterns of SnO_2_ nanoparticle powder sample, SnO_2_ photoanode, SnO_2_ photoanode after TiO_x_ post-treatment, SnO_2_ photoanode after photoanalysis process, and SnO_2_ photoanode with TiO_x_ post-treatment after photoanalysis process. Black bars show peak positions of rutile SnO_2_ structure of JCPDS card No. 00-041-1445 and red bars shows peak positions of TiO_2_ rutile structure of JCPDS card no. 01-088-1175

**2. X-ray photoelectron spectroscopy analysis (XPS)**

XPS Analysis was performed using a VG Escalab 220i-XL equipped with an Al source. The XPS data were analyzed by using CasaXPS software. Shirly subtraction is used for background correction^1^ and then the shape of the peaks used for the deconvolution is Gaussian-Lorentzian shape. Figure S2 shows the high resolution XPS of Ti 2p of the SnO_2_ photoanode after TiO_x_ post-treatment and the SnO_2_ photoanode after TiO_x_ post-treatmentafter photoanalysis process. There is no significant change for Ti^4+^ after the cell operation.


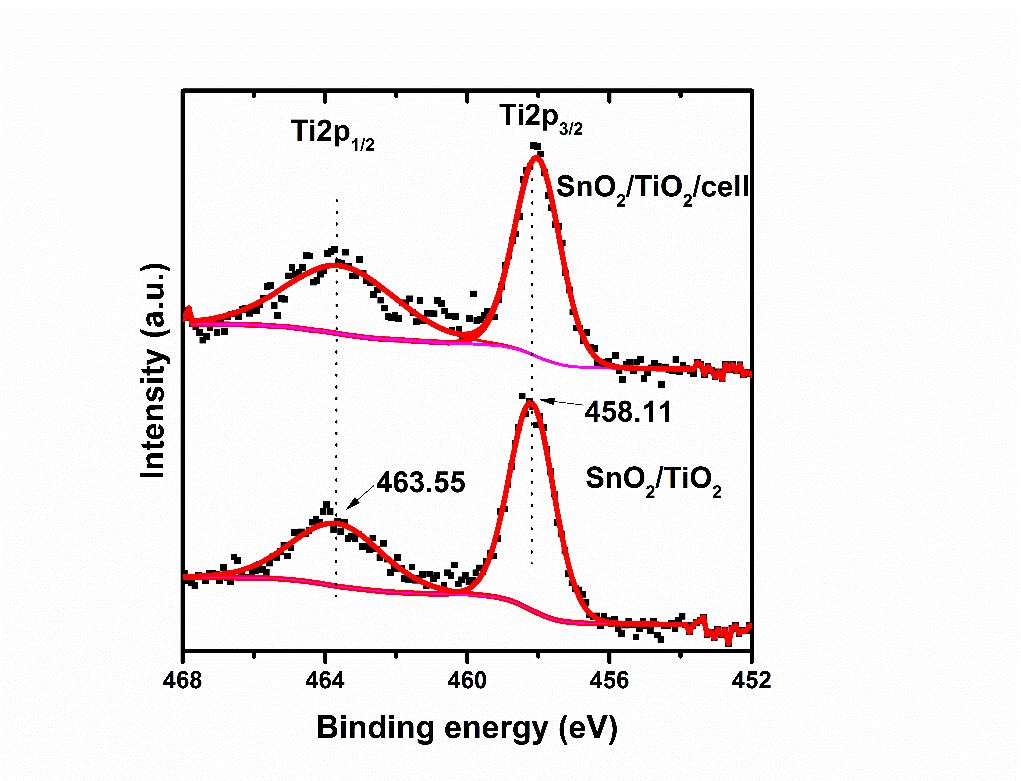


Figure S2: High-resolution X-ray Photoelectron peak spectra and curve fit of Ti 2p for SnO_2_ photoanode after treatment, SnO_2_ photoanode after photoanalysis process, and SnO_2_ photoanode with treatment after photoanalysis process.

In Figure S3, we report the Sn 3d peaks for SnO_2_ photoanode, SnO_2_ photoanode after treatment, SnO_2_ photoanode after photoanalysis process, and the treated anode after photoanalysis process. The XPS clearly reveals that there is no significant change in the Sn^4+^ state after the post treatment and also after cell fabrication and photoanalysis process. Since there is no significant shift in the Sn 3d peak position, so, from our XPS results we confirm that there is no oxidation/reduction of SnO_2_.


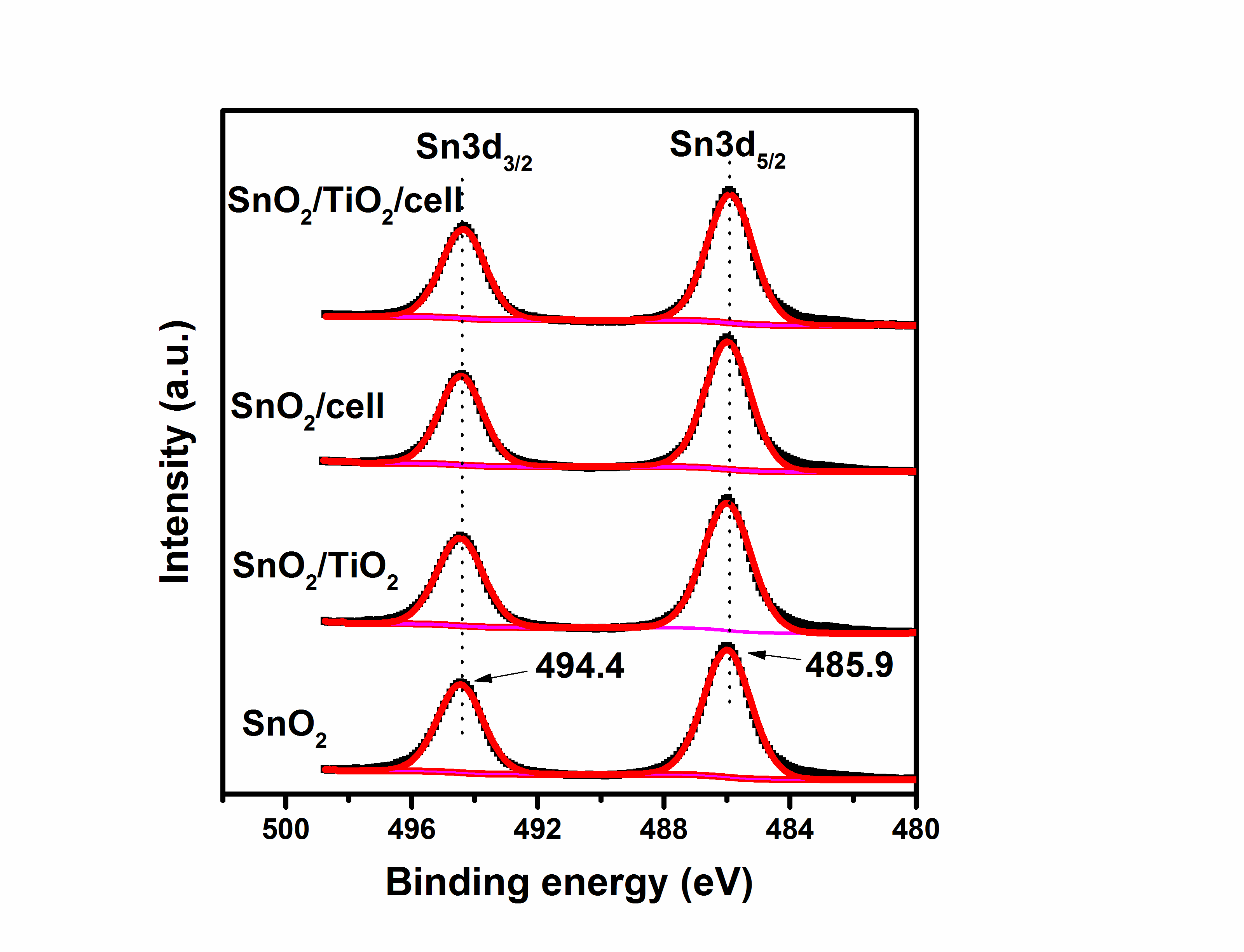


Figure S3: High-resolution X-ray Photoelectron peak spectra and curve fit of Sn 3d for SnO_2_ photoanode, SnO_2_ photoanode after treatment, SnO_2_ photoanode after photoanalysis process, and the treated anode after photoanalysis process

**Reference**

1.
